# A multiscale imaging and modelling dataset of the human inner ear

**DOI:** 10.1038/sdata.2017.132

**Published:** 2017-09-19

**Authors:** Nicolas Gerber, Mauricio Reyes, Livia Barazzetti, Hans Martin Kjer, Sergio Vera, Martin Stauber, Pavel Mistrik, Mario Ceresa, Nerea Mangado, Wilhelm Wimmer, Thomas Stark, Rasmus R. Paulsen, Stefan Weber, Marco Caversaccio, Miguel A. González Ballester

**Affiliations:** 1 ARTORG Center for Biomedical Engineering Research, University of Bern, Switzerland; 2 Institute for Surgical Technology and Biomechanics, University of Bern, Bern 3100, Switzerland; 3 Technical University of Denmark, Copenhagen 2800, Denmark; 4 Alma IT Systems, Barcelona 8007, Spain; 5 Scanco Medical AG, Brüttisellen 8306, Switzerland; 6 Med-El, Innsbruck 6020, Austria; 7 Universitat Pompeu Fabra, Barcelona 8007, Spain; 8 Department of Otorhinolaryngology, Technical University Munich, Munich 80333, Germany; 9 Department of ENT, Head and Neck Surgery, Inselspital, University Hospital of Bern, Bern 3100, Switzerland; 10 ICREA, Barcelona 8007, Spain

**Keywords:** Anatomy, Biomedical engineering

## Abstract

Understanding the human inner ear anatomy and its internal structures is paramount to advance hearing implant technology. While the emergence of imaging devices allowed researchers to improve understanding of intracochlear structures, the difficulties to collect appropriate data has resulted in studies conducted with few samples. To assist the cochlear research community, a large collection of human temporal bone images is being made available. This data descriptor, therefore, describes a rich set of image volumes acquired using cone beam computed tomography and micro-CT modalities, accompanied by manual delineations of the cochlea and sub-compartments, a statistical shape model encoding its anatomical variability, and data for electrode insertion and electrical simulations. This data makes an important asset for future studies in need of high-resolution data and related statistical data objects of the cochlea used to leverage scientific hypotheses. It is of relevance to anatomists, audiologists, computer scientists in the different domains of image analysis, computer simulations, imaging formation, and for biomedical engineers designing new strategies for cochlear implantations, electrode design, and others.

## Background & Summary

The anatomy of the human cochlea is subject to research in many areas of scientific and technological development including audiological studies, the development of less invasive surgical procedures and the design of more effective artificial hearing implants. Since the emergence of imaging technologies, it has become evident that imaging of the human cochlea has played a central role in these and other areas of research^1–4^. In audiology, the intrinsic relation between cochlear function and shape has been exhaustively studied, and has enabled the use of imaging information to develop functional models^5–7^. Similarly, in image-guided surgery of cochlear interventions, imaging has allowed researchers to study safety margins and how the anatomical variability is to be taken into account for safer and less invasive surgical procedures^8–12^. In electrophysiological modelling of the human cochlea, initial works have used synthetic and simplistic models, and despite the awareness on the importance of using realistic models generated from patient data, the difficulties to collect appropriate data has resulted in studies conducted with few samples^13–15^. In relation to the anatomical size of the human cochlea and its internal structures, the geometric resolution of current clinical computed tomography (CT) scans is considerably low. As a result, images acquired from patients typically lack information on the intracochlear anatomy and are therefore of limited usage for precise and accurate patient treatment or for the improvement and development of artificial hearing implants. To counter the limited resolution of clinical CT imaging, several attempts have been proposed to estimate the information of interest (e.g., complete cochlear duct length, position of the basilar membrane) from surrogate data measured or derived from CT images^16–19^. However, the complexity of the cochlear anatomy lowers the effectiveness of these approaches, as the observable surrogate measures are not capable of fully characterizing the internal cochlear anatomy.

With the advent of modern imaging techniques, such as micro computed tomography (μCT), the possibility to obtain detailed imaging information has allowed researchers to capture details of the cochlear anatomy that were not possible before. The current limitations of this technology for clinical integration are the reduced size of the scanning field of view, and the high amount of radiation dose required to obtain high level of image quality. However, ex-vivo studies using μCT imaging information have enabled researchers to validate important scientific hypotheses and computational models of the human cochlear physiology^1,20–23^.

Solutions to make connections between the relatively ‘low-resolution’ clinical scenario and the ex-vivo μCT imaging have emerged in recent years through the development of advanced computational models that use μCT information to build high resolution models of the cochlear anatomy, which can be used to infer the patient-specific anatomy from the low-resolution clinical CT image^24^. These computational models are able to better capture the three-dimensional correlations between shape information derived from CT and μCT imaging.

Construction of these high-resolution models requires the application of image processing techniques on high-resolution imaging. In addition to the high resource demands of μCT imaging, the release of this data descriptor makes an important asset for future studies in need of high-resolution data and related models and statistical data objects of the cochlea used to leverage scientific hypotheses.

This data descriptor encompasses a rich set of imaging data sets of the human cochlea, accompanied by manual delineations of the cochlea and sub-compartments, a statistical shape model encoding its anatomical variability, and models for electrode insertion simulation and electrical stimulations. It is of relevance to anatomists, audiologists, computer scientists in the different domains of image analysis, computer simulations, and imaging formation, as well as for biomedical engineers designing new strategies for cochlear implantations, electrode design, and others.

**Figure 1:**
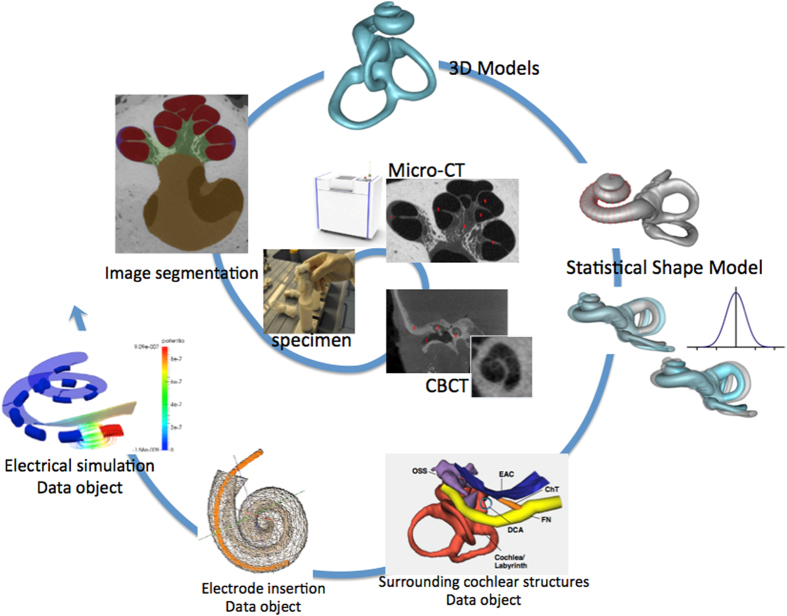
Spiral of cochlear data generation: From harvested cochlea specimens, μCT and Cone-beam CT scans, image segmentations, 3D models of the human cochlea, statistical shape model, geometrical models of surrounding cochlear structures, finite-element data objects for electrode insertion and electrical simulation. The resulting data are summarized in Tables 2 and 3.

The provided data includes 52 temporal bones scanned with clinical cone beam CT (CBCT) and μCT resulting in 30 and 50 image volumes respectively. Manual and semiautomatic segmentations of the cochlea from μCT data are provided for 24 samples. A statistical shape model (SSM) that describes the main patterns of shape variability has been built from a subset of the available data, and can be used to generate statistically plausible sample shapes programmatically. Finally, we contribute with several data objects for finite-element simulations of electrode insertion and electrical stimulation. An overview of the data generation can be seen in Fig. 1.

## Methods

In the following sections, detailed description of the samples preparation, image acquisition and mesh modelling is presented. Since the database encompasses different modalities and protocols, the methods are separated per dataset when appropriate, following the convention in Table 1.

All specimens in this data descriptor are coming from the University of Bern and the Technical University of Munich (TUM). Approval from the local ethical body in Bern was received for the specimens included herein (Ethics Commmission of Bern, Switzerland, KEK-BE Nr. 2016-00887). Specimens from the Technical University of Munich were provided according to the World Medical Association Declaration of Helsinki^25^.

**Figure 2:**
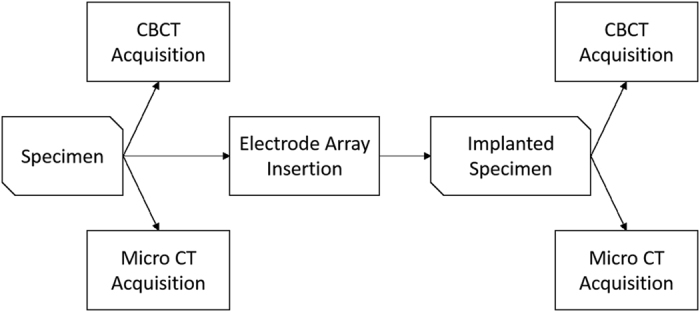


In general, the workflow depicted in Fig. 2 was followed in order to provide CBCT and μCT image datasets of the specimens. In addition, a subset of the specimens were implanted using cochlear implants electrode array and imaged for further implantation analysis.

### Human cadaver specimens preparation

All imaging and mesh data provided in this data descriptor originate from sets of cadaveric human temporal bone specimens containing the cochlea. The specimens are organized and summarized in Table 1 and described below in terms of preservation of the biological tissue, extraction and preparation, imaging methodology, image processing approaches, statistical shape modeling, and finite element model creation.

#### Specimen collection A

In total, 20 petrous temporal bones were extracted from 10 whole human cadaver head specimens preserved in Thiel solution^26,27^. The Thiel fixation method is known to preserve the mechanical properties of the tissue without the hardening and shrinkage of soft tissue, while conserving the specimen for long periods of time, similarly to formaldehyde. The specimens were prepared to fit in a sample holder with a diameter of 34 mm prior to image acquisition. While 19 petrous bones contained the complete external auditory canal, middle and inner ear, one case was damaged during the extraction process (the anterior semicircular canal was slightly cut) and was thus excluded from this descriptor.

#### Specimen collection B

Seven human cadaveric specimens were obtained from a previous study investigating a minimally invasive robotic approach for cochlear implantation^8^. A small tunnel (1.8 mm in diameter), originating on the mastoid surface and targeting the center of the round window, was drilled in each specimen and free-fitting CI electrode arrays were manually inserted. In order to allow imaging using a μCT scanner, the petrous part of the temporal bone was extracted from the whole head specimens (including the external auditory canal, the middle ear and the inner ear). A detailed description of the materials and methods is given in Bell *et al.*^8^ and Wimmer *et al.*^12^.

#### Specimen collection C

A total of 20 dry temporal bone specimens were provided by the anatomical collection of the Institute of Anatomy, University of Bern, Switzerland. No intracochlear structures such as the basilar membrane or the round/oval window membranes were preserved. Thus, only the calcified tissues are visible in the images. The sample holder size of the μCT was chosen individually per sample in order to fit the specimen size containing the complete inner ear. All specimens were fixed in the sample holders with polystyrene foam to avoid relative motion of the specimens during the scans.

#### Specimen collection D

Five petrous temporal bones were frozen and preserved at −20 °C, without additional fixation, and defrosted 3 h before scanning. Four samples were implanted with MED-EL Flex^EAS^ dummy electrode arrays excluding wires (in order to avoid metal artifacts) using a transmastoid approach and a posterior tympanotomy. The semicircular canals could not be retained in order to be able to fit the specimens to the 17 mm μCT holder of a μCT 50 scanner (Scanco Medical AG, Brüttisellen, Switzerland). A detailed description is available in^1,28^.

### Image acquisition

**Figure 3:**
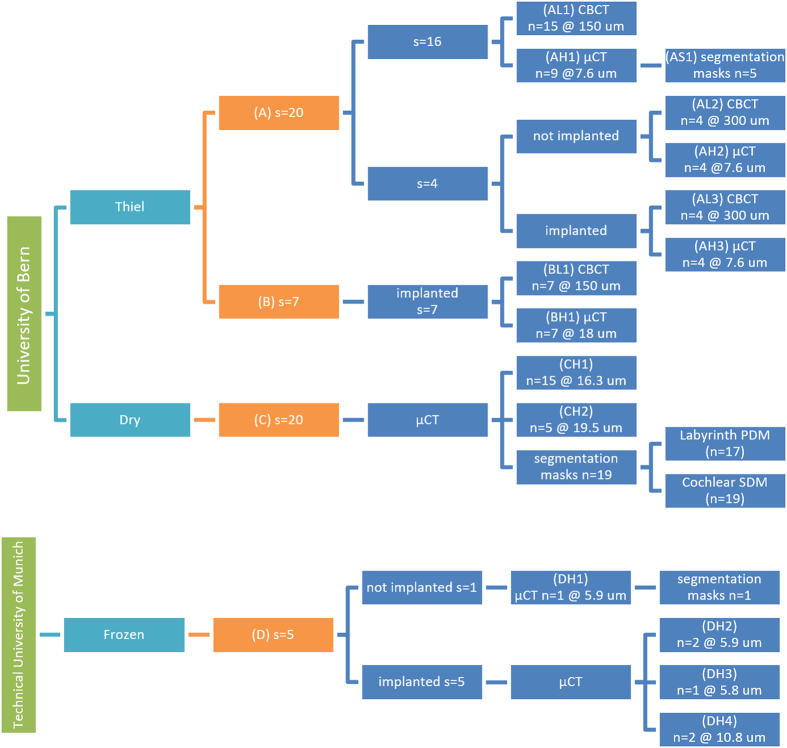
Overview and origin of acquired image datasets with number of specimens (s) and number of image volumes (n). Each image set was given an ID composed of the specimen provenance (letter **A**–**D**), low, high resolution or segmentation mask (L, H or S) and a set identification number.

Images from the prepared specimens were acquired using μCT and CBCT modalities. An overview of the resulting image datasets are depicted in Fig. 3. The diagram is organized as follows: specimen affiliation, preservation method, collections, followed by CBCT and μCT imaging for intact and implanted specimens.

#### μCT imaging

All prepared specimens from collections A and C were imaged using a μCT 100 scanner (Scanco Medical AG, Brüttisellen, Switzerland). Additionally, the prepared specimens from collection D were scanned using a μCT 50 scanner (Scanco Medical AG, Brüttisellen, Switzerland). Table 2 summarizes the μCT measurement parameters for the different specimen collections. All specimens were fixed in the sample holders with polystyrene foam to avoid relative motion of the specimen during the scans. No medium such as ethanol or phosphate-buffered saline (PBS) was added and the specimens were scanned in air to obtain the best image contrast.

The data was reconstructed using a filtered back-projection algorithm. From an initial reconstruction, a region of interest was selected and subsequently reconstructed to 4,608×4,608 pixels per slice. The resulting voxel size is 7.6 μm for the Thiel fixed specimens, 16.3 μm for the specimens measured in the 73 mm sample holder and 19.5 μm for the specimens measured in the 88 mm sample holder, respectively. Finally, the reconstructed data was converted and stored as sequences of DICOM images.

#### CBCT imaging

While μCT may provide sufficient spatial resolution to display intracochlear membranous structures, it is limited to in-vitro examinations with samples restricted in size. This data descriptor is therefore augmented with clinically applicable modalities such as the cone beam CT (CBCT). 15 specimens from collection A and 7 specimens from collection B were scanned with a ProMax 3D Max CBCT scanner (Planmeca, Finland). The specimens were placed in a plastic container at the approximate center of the revolving scanning arm. A lower skull scan protocol with the following parameters was used: 90 kVp, 8 mA, 100 mm FOV, 108 mAs and a slice thickness of 0.15 mm. The focal spot was 0.6 mm×0.6 mm according to the manufacturer’s documentation. The resulting reconstructed stack of images has an isotropic voxel size of 150 μm and are saved in a sequence of DICOM files.

In addition, 8 specimens from collection A were imaged using the xCAT® mobile CBCT scanner (Xoran Technologies, United States). The specimens were placed in a plastic container at the approximate center of the rotating gantry. A high resolution scanning protocol was used using the following parameters: 120 kVp, 6 mA, 245 mm FOV. The resulting reconstructed stack of images has an isotropic voxel size of 300 μm and are saved in a sequence of DICOM files.

#### Summary of acquired image datasets

The following table summarizes the different image datasets obtained from imaging the specimen collections.

### Micro computed tomography segmentation

#### Specimen collection A

The contrast in the images enables the distinction between cochlear fluid-filled regions, soft-tissue and bone. The following inner ear structures were manually segmented on 5 μCT image datasets using a commercially available software (Amira, FEI Visualization Sciences Group):

- The scala tympani and scala vestibuli. Because the image resolution was not sufficient to visualize the Reissner’s membrane, the scala media could not be identified and was included in the scala vestibuli segmentation.
- The vestibule and the semicircular canals
- The modiolus (including the interscalar septum)
- The lamina spiralis
- The spiral ligament
- The basilar membrane
- The round and oval window membranes

The data resulting from segmentation is a volumetric label image, where each voxel in the original volume has been assigned a label. A label is an integer value indicating the nature of the underlying tissue. Surface models of the different anatomies can be generated from the label volumes using standard iso-surface extraction techniques^29^.

#### Specimen collection C

The contrast in the set of images from collection C enables bone and non-bone structures to be distinguished. The labyrinth was segmented by using a single object/label representing the *cochlear scalae (*i.e., *scala tympani, vestibuli and media)*, *vestibule* and *semicircular canals.* The *lamina spiralis* was excluded from the object, and the openings to the oval and round window had no obvious boundaries to demarcate the segmentation. Consequently, a consistent smooth manual closing was performed in these regions. The manual closing of the oval and round window and segmentation of the cochlea, vestibule and semicircular canals was performed using the software tool ITK-SNAP^30^, while the manual corrections of the segmentations were made using the software tool Seg3D (www.seg3d.org). It resulted in 19 labelled image datasets.

#### Specimen collection D

Two image datasets were manually segmented with a commercially available software (Amira, FEI Visualization Sciences Group). In the case of the non-implanted temporal bones, the segmented structures were the scala vestibuli, scala tympani, osseous spiral lamina, round window, cochlear partition and stapes. Since the Reissner’s membrane was missing, the scala media could not be individually identified and was included in the scala vestibuli segmentation. In the case of the implanted temporal bones, the structures segmented were the electrode array, scala tympani and scala vestibuli.

### Statistical shape analysis

#### Statistical shape modeling (SSM)

is a powerful technique used to represent the anatomical variability of a given structure or organ as a compact and parametric mathematical model. The first step of the process is to establish correspondences between the samples. The principle described in Frangi *et al.*^31^ is followed, where the correspondences are found through a volumetric image registration between a chosen reference and each of the remaining available samples. Following, the image registration a mesh structure was propagated to all samples using the deformation fields computed using the volumetric registration.

**Labyrinth PDM (** **Data Citation113**): From the specimen collection C, 17 samples could be used to build a classic Point Distribution Model (PDM)^32^ of the inner ear labyrinth, with the registration model detailed in ref. 33. In short, the datasets were downsampled to 24 μm voxel-sizes and then rigidly aligned to take out variability in translation and rotation between the samples. In order to ensure a good quality of the following non-rigid registration, especially for the cochlear turns and apical region, a simple model of the cochlea skeleton was introduced, which provides a more suited way of describing the similarity between two cochlea samples. The non-rigid multi-level cubic B-spline registrations were made following the framework and formulation of the elastix software library^34^.

**Cochlea PDM (** **Data Citation114**): The reference 3D surface model is projected to each of the individual samples through the image registration transformations. A classic linear principal component analysis (PCA) is made using Statismo^35^. The result is a point distribution model (PDM) that models the variation in the surface point coordinates.

### Virtual electrode array insertion and finite element mesh generation

In order to generate a complete computational model of the cochlear implantation, the electrode array needs to be virtually implanted into the specific cochlear anatomy, previously created. Thus, a virtual insertion is first performed followed by the generation of a volumetric finite element mesh of the whole model allowing further assessment of the electrical activation using Finite Element Methods (FEM).

During the virtual insertion step, first, the vestibular and semicircular canals are removed from the virtual cochlea and an insertion point is estimated at the center of the round window membrane. A specific electrode array is subsequently generated from a parametric model that describes the possible shape, size, number and type of electrode contacts using template files written in the open-source CAD language OpenSCAD (http://www.openscad.org). Once both the processed cochlea and specific selected electrode are defined, the insertion of the electrode inside the virtual ear is performed. A set of issues need to be considered to compute a virtual insertion. There exist many possible surgical trajectories and the final position of the implant during a real intervention will depend on several factors such as the stiffness of the electrode itself, the dexterity of the surgeon and the interaction between the insertion tools and the patient’s anatomy.

Additionally, to reduce computational complexity, the virtual insertion step is optimized for potential repeated use. A two-step solution is proposed. First computing a possible surgical trajectory using the SOFA framework, using simplified geometrical models and greedy collision detection algorithms, and second deforming the shape of the original electrode design in the new position using the parallel transport frame^36,37^. The collision model for the electrode array is generated using a set of points and lines along the centerline of the object for reducing computation time^38,39^. Several trajectories are pre-computed to speed up the process accommodating the most common scenarios, but can be traced again for special cases. Overall, this results in a very flexible and lightweight approach to control the insertion of the electrode into the cochlea.

**Figure 4:**
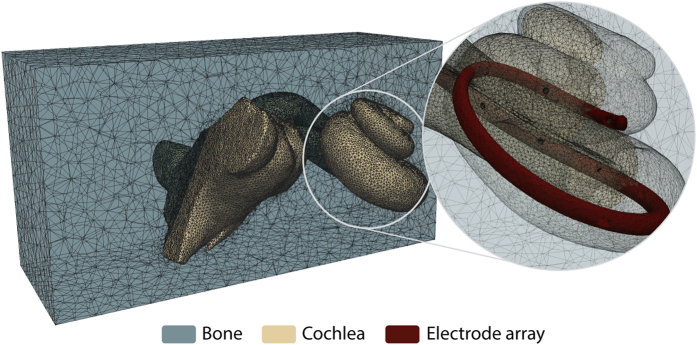
Finite element mesh obtained for a single patient. Faces are cut for visualization purpose.

Once the virtual insertion is completed, the generation of the volumetric mesh is performed. This mesh is used to carry out the stimulation of the electrical activation of the nerve fibers due to the implant activation. Starting from the surface models of the virtually implanted cochlea, the nerve fibers are automatically generated considering the position of the spiral ganglion and a surrounding temporal bone, which is crucial for a realistic propagation of the stimulating currents. Spheres of 0.75 mm of diameter are created on the electrode's contact to ease the definition of boundary conditions for the electrical stimulation. Afterwards, all the structures described so far are merged and a single tetrahedral mesh is generated (see Fig. 4) and proper FEM definitions are applied. Further details of the framework can be found in Mangado *et al.*^40^ and a full application to the case of patients with healthy and degenerated nerve auditory fibers in Ceresa *et al.*^41^. The aspect ratio of all elements contained in the final mesh is computed to quantify the mesh quality of the model, in order to avoid convergence problems during the finite element simulation. Finally, the tetrahedral mesh is exported into a GMESH2 file format (MSH) which details the list of tetrahedral elements and their connections^42^.

### Code availability

#### Statismo framework

Statismo is an open source C^++^ framework for statistical shape modeling^43^. It supports all shape modeling tasks, from model building to shape analysis. Although the focus of Statismo lies on shape modeling, it is designed such that it supports a variety of statistical models, including statistical deformation models and intensity models. One of the main goals of Statismo is to make the exchange of statistical shape models easy. This is achieved by using a well-documented file format based on HDF5. https://github.com/statismo/statismo.

The following software tools were used for manual segmentation and manual correction of the segmentation masks:

#### ITKSnap

The manual brushing tool was used to alter the manual segmentations^30^.http://www.itksnap.org/Used Version: 2.4

#### Seg3D

The manual brushing tool was used to alter the manual segmentations^31^.http://www.sci.utah.edu/cibc-software/seg3d.htmlUsed Version: 2.14

For general-purpose tasks, such as cropping and reformatting recommended open source software include Slicer3D (www.slicer.org)^44^ and MevisLab (www.mevislab.de)^45^.

## Data Records

All data records described in this manuscript are available on the SICAS Medical Image Repository (www.smir.ch)^46^ (Data Citation 1 to Data Citation 112) organized in virtual folders, each describing the data provenance and modality. Computed tomography three-dimensional files are stored using the Digital Imaging and Communications in Medicine image file format (DICOM, ISO 12052). A three-dimensional volume is physically stored as a stack of single sliced images. Data Citation 113 and Data Citation 114 correspond to the statistical shape models of the cochlear labyrinth (C-PDM) and the cochlear structure only (C-SDM), respectively.

## Technical Validation

### Image acquisition

μCT datasets were acquired using the two commercial μCT systems; μCT 50 and μCT 100, Scanco Medical AG, Brüttisellen, Switzerland. These systems are delivered with phantoms to calibrate and verify the geometry and the density response of the systems.

To verify the geometry, a thin wire is measured and its volume is quantified. If the geometry changes, the volume gets out of a given range and the cross-section of the wire is no longer a circle. In this case, the geometry has to be re-calibrated. This phantom has been measured monthly as recommended by the manufacturer. In this study, no geometry re-calibration was required.

To verify the density response of the μCT systems, a phantom including five cylinders with known densities in a range from 0 to 800 mgHA/ccm is measured. If the density response is altered, the values are out of a given range and the system has to be re-calibrated. Furthermore, the density of the cylinders can be used to calibrate the grey levels to bone density values in the given range. This phantom has been measured weekly as recommended by the manufacturer. In this study, no density re-calibration was required.

The actual validation of each μCT image was done visually by an experienced user. Image quality was checked for consistency, artifacts and image quality. Every single measurement was visually checked and if quality was considered not to be sufficient, the measurement has been repeated.

### Data segmentation

A neuroradiologist reviewed the segmentation datasets to account for anatomical malformations. The following image artefacts or anatomical malformations were observed in the datasets hindering the segmentation process:

- Drilling trajectories appear on the datasets from collection B. These drillings come from experiments performed as part of a previous study on cochlear implantation^8^.
- Some areas show deviations from the normal anatomy, possibly caused by anatomical variations or debris coming from the fixation process or subsequent robotic drilling.
- The round and oval window membranes are in most of the cases lost or partially lost in the images. Missing window membranes hinder the segmentation process due to loss of connectivity.

## Usage Notes

To process the provided images, it is highly recommended to use medical image tools which handle consistently the physical space and orientation of the images. We verified that all the used formats (DICOM, Nifti, Metaimage), the segmentations and the meshes can be loaded correctly with 3D Slicer (www.slicer.org)^44^.

The statistical shape model is an HDF5 file which respects Statismo format. It is possible to view the mean shape and extract samples with the Statismo viewer or the Statismo CLI^47^. The deformation fields can be applied to the reference to obtain varying samples^34^. The later requires an integration between elastix and Statismo as available at https://github.com/tom-albrecht/statismo-elastix.

## Additional Information

**How to cite this article:** Gerber, N. *et al.* A multiscale imaging and modelling dataset of the human inner ear. *Sci. Data* 4:170132 doi: 10.1038/sdata.2017.132 (2017).

**Publisher’s note:** Springer Nature remains neutral with regard to jurisdictional claims in published maps and institutional affiliations.

## Supplementary Material



## Figures and Tables

**Table 1:** Summary of human cadaver specimen collections.

**ID**	**Source**	**Specimen**	**Preservation Method**	**Number of Samples**
A	University of Bern	Petrous bone	Thiel	19
B	University of Bern	Petrous bone	Thiel	7
C	University of Bern	Petrous bone	Dry	20
D	Technical University of Munich (TUM)	Cochlea	Frozen	5

**Table 2:** μCT measurement settings used for the different collections of specimens.

**Sample Holder Diameter**	**TUM (*****n*****=4) 17 mm**	**TUM (*****n*****=2) 32 mm**	**Thiel (*****n*****=16) 34 mm**	**Dry (*****n*****=15) 73 mm**	**Dry (*****n*****=5) 88 mm**
Energy	70 kVp	70 kVp	90 kVp	90 kVp	90 kVp
Intensity	200 μA	200 μA	88 μA	155 μA	155 μA
Filtering	Al 0.5 mm	Al 0.5 mm	Al 0.5 mm	Al 0.5 mm	Al 0.5 mm
Integration time	800/1,000 ms	800/1,000 ms	600 ms	350 ms	350 ms
Frame averaging	4 x	4 x	2 x	4 x	4 x
Samples	3,400	3,400	3,072	3,072	3,072
No. projections/180°	1,500	1,500	2,000	2,000	2,000
HR reconstruction voxel size	5.9 μm	10.8 μm	7.6 μm	16.3 μm	19.5 μm
Average measurement time	36 h	36 h	25.5 h	23.4 h	21.0 h

**Table 3:** Summary of acquired image datasets

**ID**	**Data Citation**	**Specimen**	**Description**	**Spatial Resolution (isotropic)**	**Number of Scanned Volumes**
CBCT Image Datasets					
AL1	1–15	Petrous bone	not implanted	150 μm	15
AL2	16–19	Petrous bone	not implanted	300 μm	4
AL3	20–23	Petrous bone	implanted	300 μm	4
BL1	24–30	Petrous bone	implanted	150 μm	7
μCT Image Datasets					
AH1	31–45	Petrous bone	not implanted	7.6 μm	9
AH2	46–49	Petrous bone	not implanted	7.6 μm	4
AH3	50–53	Petrous bone	implanted	7.6 μm	4
BH1	54–60	Petrous bone	implanted	18 μm	7
CH1	61–75	Petrous bone	not implanted	16.3 μm	15
CH2	76–80	Petrous bone	not implanted	19.5 μm	5
DH1	81	Cochlea	not implanted	5.9 μm	1
DH2	82–83	Cochlea	implanted	5.9 μm	2
DH3	84	Cochlea	implanted	5.8 μm	1
DH4	85	Cochlea	implanted	10.8 μm	2
AS1	86–90	Petrous bone	Segmentation label	7.6 μm	5
CS1	91–103	Petrous bone	Segmentation label	16.3 μm	13
CS2	104–107	Petrous bone	Segmentation label	19.5 μm	4
DS1	108	Cochlea	Segmentation label	5.9 μm	1
DS2	109	Cochlea	Segmentation label	5.9 μm	1
DS3	110	Cochlea	Segmentation label	5.8 μm	1
DS4	111–112	Cochlea	Segmentation label	10.8 μm	2
The image dataset ID is composed of the provenance (letter A-D), low, high resolution or segmentation mask (L, H or S) and a set identification number.					
